# Age-dependent regulation of host seeking in *Anopheles coluzzii*

**DOI:** 10.1038/s41598-019-46220-w

**Published:** 2019-07-04

**Authors:** A. B. Omondi, M. Ghaninia, M. Dawit, T. Svensson, R. Ignell

**Affiliations:** 1 Disease vector group, Unit of Chemical Ecology, Department of Plant Protection Biology, Swedish University of Agricultural Sciences. Sundvägen 16, SE-23053, Alnarp, Sweden; 2 School of Life Sciences, Arizona State University, Tempe, AZ 85287 USA; 3 Division of Entomology, Department of Plant Protection, Gorgan University of Agricultural Sciences and Natural Resources, Grogan, Iran; 4 Department of Zoological Sciences, Addis Ababa University, P. O. Box 1176, Addis Ababa, Ethiopia; 5 Present Address: Bioversity International, IITA Campus, Abomey Calavi, 08 BP 0932, Cotonou, Benin

**Keywords:** Behavioural ecology, Chemical ecology

## Abstract

Behavioural attraction of the malaria vector *Anopheles coluzzii* to human host odour increases during adult maturation. We have previously demonstrated that the onset of host seeking in *An. coluzzii* coincides with an increased sensitivity of the CO_2_-sensitive neurons and abundance of chemosensory receptor gene transcripts in the maxillary palp. In this study, we extend our analysis to the antenna. Functional characterisation of the near-complete repertoire of odorant receptors (Ors) expressed in this tissue, to fractioned human odour, reveals a subset of salient human odorants to be detected by Ors at physiological relevant concentrations. When presented as a blend in their ratio of natural emission, these odorants elicit attraction by host-seeking mosquitoes, emphasising that Ors alone can mediate this behaviour. However, the same blend inhibits attraction in teneral mosquitoes. This switch in behavioural response indicates a change in valence during adult maturation. Quantitative analysis of *Or* transcript abundance and *in vivo* electrophysiological analysis reveal that the olfactory system of female *An. coluzzii* undergoes concerted changes that correlate with the onset of host seeking. We conclude that changes in *Or* abundance modulate peripheral olfactory coding, resulting in ecologically relevant behavioural effects.

## Introduction

Blood-feeding female mosquitoes, including the malaria vector *Anopheles coluzzii* (previously *Anopheles gambiae* M molecular form), gradually increase their behavioural response to human host odour during adult maturation^1–5^. While the teneral (freshly emerged) stage of blood-feeding mosquitoes actively search for sugar-rich resources to supplement their nutrient reserves^6^, the capacity to seek a human host does not develop until 24–72 h after adult eclosion^2–4^. The reason for this is that teneral females do not have appropriately developed mouthparts to enable them to take a blood meal^7^. This plasticity ensures an appropriate, context-dependent response that allows female mosquitoes to engage in host seeking at times when they require blood for egg development. Elucidating the molecular basis of this age-dependent plasticity could reveal novel targets for vector control.

The onset of host seeking in female mosquitoes coincides with an increase in sensitivity of the olfactory sensory neurons (OSNs) on the antennae and maxillary palps to salient human odorants^1–3,5,8^. Adult maturation in *An. coluzzii* and the dengue vector, *Aedes aegypti*, leads to an increased sensitivity to carbon dioxide (CO_2_)^1,5^. Emitted by all vertebrates, CO_2_ elicits activation and attraction, and gates the behavioural response to other human odorants^8–10^. In *Ae. aegypti*, the OSN sensitivity to lactic acid and (*R*)-1-octen-3-ol, two odorants important for contextual host recognition, also increases with adult maturation^1,3,11,12^. This change in sensory sensitivity correlate with an increased behavioural response to these host odorants^1,3^. Recent studies have also revealed a functional connection between the increased sensory and behavioural response, and the increased transcript abundance of chemosensory genes encoding for the receptors tuned to CO_2_ and (*R*)-1-octen-3-ol in *An. coluzzii* and *Ae. aegypti*^1,5^. This suggests a direct relationship between the transcription of select chemosensory receptor genes and the function of the gene products and their cognate ligands, which translates into behaviours that are regulated by the physiological condition of the insects.

The odorant specificity and sensitivity of most OSNs in mosquitoes are conferred by the expression of odorant receptor (Or) genes^13–21^. Both the targeted mutation of *Orco*, encoding for the obligate co-receptor Orco, and the differential expression of a variable *Or* in *Ae. aegypti*^14,18^ have shown that Ors are critical for accurate host recognition. However, their involvement in host attraction is still to be confirmed^22^. The functional classification of the Or repertoire in *An. coluzzii*, encompassing more than 50 genes in the adult mosquito^19,20,23^, has been a monumental step towards the characterisation of odorant reception in malaria mosquitoes^13,21,24^. However, the tuning of the *An. coluzzii* Ors has not been characterised using ecologically relevant sources and doses of odorants, including the fractioned odour from humans, which encompass several hundreds of volatile organic compounds^25–27^. Such an ecologically focused approach is pertinent for the identification of odorants found in the natural environment of the mosquito, and of their corresponding signalling pathways^12,18^.

Here, we expand our analysis of the age-dependent plasticity in the olfactory system of *An. coluzzii*. We identify salient human odorants, detected by a limited array of Ors, which when presented as a blend in their natural emission ratios, elicit differential behavioural responses in teneral and host-seeking female mosquitoes. Transcription analysis, together with electrophysiological and behavioural analyses, indicates distinct Or-signalling pathways to be involved in the regulation of the age-dependent response to human odour.

## Results

### The Or repertoire responds to a small subset of human odorants

**Figure 1:**
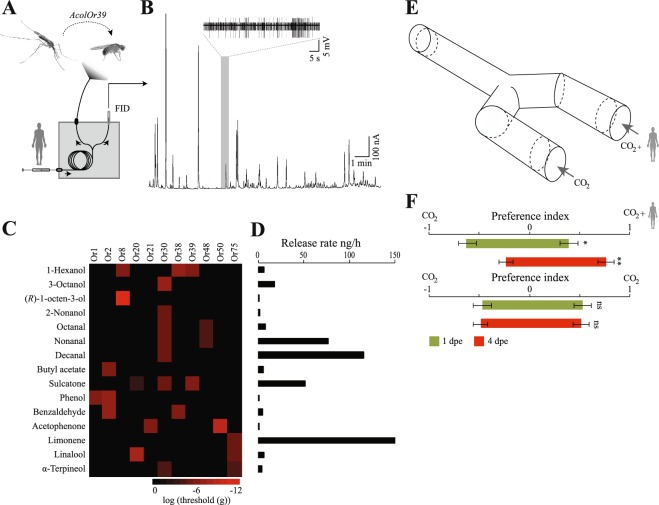
Select Ors of *Anopheles coluzzii* respond to human odorants, which when combined elicit differential behavioural responses in teneral and host-seeking female mosquitoes. (**A**) Schematic of the combined gas chromatography-coupled single sensillum recording (GC–SSR) setup. FID, flame ionization detector. (**B**) Sample GC-SSR trace, with the SSR trace at the top showing the response of the Or39-expressing neuron (large amplitude neuron) and the FID chromatogram showing the elution of human odorants. (**C**) Heat map displaying the threshold of response of the Ors responding to human odorants. (**D**) Release rate of the human odorants. (**E**) Y-tube olfactometer design (synthetic human odour with CO_2_ versus CO_2_). (**F**) Behavioural responses of teneral (1 day post-eclosion (1 dpe)) (green) and host-seeking (4 dpe) (red) mosquitoes to the full synthetic human odour blend + CO_2_ versus CO_2_ (top), and to CO_2_ versus CO_2_ (bottom). Statistical significance was tested using nominal logistic regression. Error bars represent standard errors of the mean. *p < 0.05, **p < 0.01, ns: not significant.

To identify human odorants detected by *An. coluzzii* Ors, we transgenically expressed 49 *Or*s individually in an OSN of *Drosophila melanogaster* lacking a variable Or^13,28^. Forty three of these *Or*s have previously been shown to be detected in the antenna and maxillary palp of *An coluzzii*, above the background level of abundance^19,20,23^. Subsequently, we analysed the response of the Ors to fractioned human odour, using combined gas chromatography and single sensillum recording (GC-SSR) analysis (Fig. 1A,B). In all, 15 compounds were found to elicit consistent responses across 11 of the 49 tested ORs (Fig. 1C). The overall volatile release rate was 443,8 ng h^−1^, with limonene, decanal and nonanal as the most abundant compounds (Fig. 1D). Individual odorants activated either a single or a subset of Ors, and individual Ors similarly responded to either a single or a subset of odorants (Fig. 1C). Receptors differed in their sensitivity to the tested odorants, as determined by SSR analysis of the ectopically expressed Ors, challenged with synthetic standards at various doses, and visualised by a heat plot indicating their threshold of response (Fig. 1C).

### Age-dependent behavioural responses to human odour

We next used a Y-tube olfactometer (Fig. 1E) to evaluate the behavioural responses of teneral (1 day post-eclosion (dpe)) and host-seeking (4 dpe) female mosquitoes to a synthetic blend of all 15 compounds, presented at their natural ratios (Fig. 1D), and embedded within a background of pulsed CO_2_, used to activate the mosquitoes. While host-seeking mosquitoes strongly preferred to enter the arm containing the blend of human odorants in the presence of CO_2_, teneral females preferred to enter the control arm, containing CO_2_ alone (Fig. 1F; upper graph). Control experiments, using pulsed CO_2_ in both arms, revealed no left-right bias within the olfactometer (Fig. 1F; lower graph).

### Age-dependent changes in or gene regulation and *in vivo* neural response

**Figure 2:**
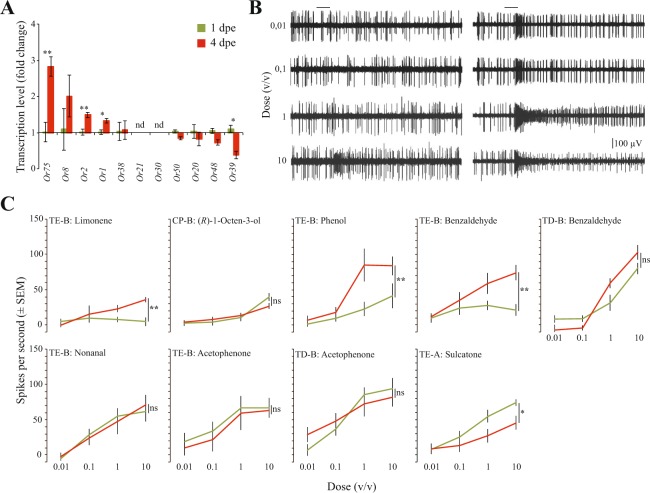
Changes in *Or* transcript abundance and sensitivity of OSNs to salient human odorants in teneral and host-seeking *Anopheles coluzzii*. (**A**) Relative transcription levels of genes encoding Ors tuned to human odorants in teneral (1 day post-eclosion (1 dpe)) (green) and host-seeking (4 dpe) (red) females. Statistical significance was tested using a two-tailed paired Student’s t-test. Error bars represent standard errors of the mean. *p < 0.05, **p < 0.01, ns: not significant. nd: not detected. (**B**) Responses of the TE-B neuron to increasing doses of phenol in teneral (left) and host-seeking (right) mosquitoes. (**C**) Physiological response of OSNs in TD, TE and capitate peg sensilla, in teneral (green) and host-seeking (red) mosquitoes, to increasing doses of human volatiles. Statistical significance was tested using a repeated measures ANOVA. Error bars represent standard errors of the mean. *p < 0.05, **p < 0.01, ns: not significant.

Real time qPCR assays revealed a significant change in the abundance of select *Or* transcripts in 4 dpe compared to 1 dpe mosquitoes (Supplementary Fig. 1; Fig. 2A). Of the ten *Or* transcripts demonstrated to be significantly more abundant in 4 dpe mosquitoes, three (*Or1*, *Or2* and *Or75*) encode for receptors that detect salient human odorants (Figs 1C, 2). Only one (*Or39*) of the 5 *Or* transcripts found to have a significantly lower abundance in 4 dpe compared to 1 dpe mosquitoes, respond to volatile compounds present in the human odour (Fig. 1C).

Single sensillum recordings from the three, previously functionally characterised^29^, morphological types of trichoid sensilla, TC, TE and TD^29^, on the antennae of 1 dpe and 4 dpe mosquitoes demonstrated differential regulation of OSN sensitivity with age, to a select number of the salient human odorants (Fig. 2B,C). In all, three functional types of antennal OSNs responded to the tested volatile organic compounds; two neurons co-localised within TE sensilla, TE-A and TE-B, as well as the B neuron housed in a TD sensillum. The sensitivity of the TE-B neuron to limonene, phenol and benzaldehyde was found to be significantly increased in 4 dpe mosquitoes compared to 1 dpe mosquitoes (Fig. 2C), whereas the sensitivity of the TE-A neuron to sulcatone was lower in the older mosquitoes (Fig. 2C). No age-related differences in sensitivity to benzaldehyde (TD-B neurons), nonanal (TE-B) or acetophenone (TE-B and TD-B, respectively) were found. Previous analysis of the capitate peg sensilla on the maxillary palp of *An. coluzzii* also revealed no difference in sensitivity to (*R*)−1-octen-3-ol (Fig. 2C; data from Omondi *et al*.^5^).

### Subtraction of salient odorants affects host-seeking behaviour

**Figure 3:**
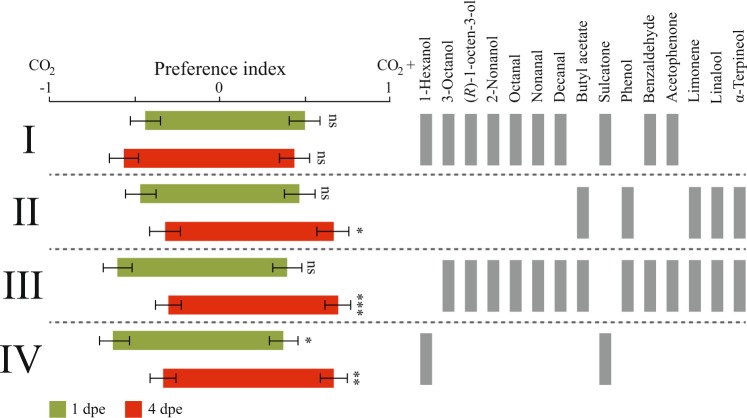
Behavioural responses of teneral and host-seeking *Anopheles coluzzii* to subtractive blends of the synthetic human odour. Removal of odorants from the full synthetic human odour blend, as indicated by the absence of grey bars, elicit differential behavioural responses in teneral (1 day post-eclosion (1 dpe); green) and host-seeking (4 dpe; red) mosquitoes. A total of four subtractive blends (I–IV) were evaluated. Statistical significance was tested using nominal logistic regression. Error bars represent standard errors of the mean. *p < 0.05, **p < 0.01, ***p < 0,001, ns: not significant.

To assess how salient human odorants, predominantly detected by the Ors displaying age-dependent regulation in gene abundance, contribute to the behavioural response of teneral and host-seeking females, we next assayed 1 dpe and 4 dpe mosquitoes to subtractive blends of the human odour, in the presence of CO_2_. We first removed the odorants detected by Or1, Or2 and Or75, for which the transcript abundance was higher in 4 dpe compared to 1 dpe mosquitoes, from the full synthetic human odour blend. This resulted in the removal of preference of 1 dpe and 4 dpe females for the control and human blend, respectively (Fig. 3; blend I). A subtractive blend, including only the compounds detected by these Ors, attracted 4 dpe mosquitoes, whereas 1 dpe mosquitoes displayed no preference for neither the blend nor the CO_2_ control (Fig. 3; blend II). In the next experiment, we removed the compounds detected by Or39, for which the transcript abundance was significantly decreased in 4 dpe compared to 1 dpe mosquitoes, from the full synthetic human odour blend. This blend elicited attraction of 4 dpe mosquitoes, whereas 1 dpe females displayed no preference for neither treatment nor control (Fig. 3; blend III). When these two compounds were tested alone, 1 dpe mosquitoes preferred the control arm, whereas 4 dpe females were attracted by this two-component blend (Fig. 3; blend IV).

## Discussion

Female mosquitoes, including *An. coluzzii*, display a context-dependent behavioural response to human host odour, directly linked to their capacity to blood feed^2–4^. By screening the near-complete repertoire of Ors expressed in adult female *An. coluzzii*^19,20,23^ using fractioned human odour, we identified salient human odorants, which, when combined in their natural ratio, reproduce these age-dependent behavioural responses. Analyses of *Or* transcript abundance and OSN activity *in vivo*, in combination with an extended behavioural analysis, suggest a direct role for specific Or-signalling pathways for salient odorant information, regulating the contrasting behavioural responses of teneral and host-seeking mosquitoes.

### The chemistry of attraction

Human odour encompasses several hundreds of volatile organic compounds^25–27^. This complexity has made it difficult to identify the behaviourally active odorants that contribute to host attraction and preference in mosquitoes. A major reason for this is that host recognition in mosquitoes is contextual, *i.e*. both quantitative and qualitative variations in host odour blends are key for eliciting olfactory codes in the olfactory system to enable host seeking and discrimination^11,12,22,30–32^. While most of the described behavioural attractants for mosquitoes, including ammonia and carboxylic acids, have been identified as, or are assumed, ligands for ionotropic receptors^11,33–36^, targeted mutation of *Orco* has shown that Ors are critical for accurate host determination^14^. In this study, we identify Ors that sensitize the antennae and maxillary palps of *An. coluzzii* to salient human odorants, which when combined in a blend elicits robust and contrasting age-dependent behavioural responses. This reveals an important role for Ors also in the regulation of behavioural attraction.

Our analysis of the Or repertoire of female *An. coluzzii* revealed that only 15 out of the hundreds of compounds present in human body emanations elicit a response in a subset of the Ors. Several of these compounds were not included in the panel used by Carey *et al*.^13^, emphasising the efficiency of the combined GC-SSR approach to identify salint odorants. Most of the compounds identified in this study are consistent markers in human skin and axillary odour^25–27,37–39^. While the skin microbiota is thought to play a pivotal role in the attractiveness of humans to malaria mosquitoes^40,41^, it is interesting that only a single compound identified in this study, butyl acetate, can be linked to the human bacterial flora^42^. Of the most abundant compounds identified in this study, the aldehydes are formed by oxidative degradation of skin surface lipids^39^, but are also produced by red blood cells along with terpenoids^43^. The release of these compounds in red blood cells is enhanced following infection with *Plasmodium falciparum* malaria parasites, resulting in an increased attraction of *An. coluzzii*^43^. Besides these compounds, only sulcatone and (*R*)-1-octen-3-ol, of the compounds identified in this study, have been shown to provide host recognition cues for *An. coluzzii* and other mosquitoes^12,18^.

Previous behavioural analyses have shown that mosquito olfaction is highly contextual, and that the behavioural attraction triggered by an odour blend is stronger than that triggered by an individual compound^12,16,30,32^. Integration of odour blends in the olfactory system has an emergent property, which leads to behavioural responses that are different from those elicited by individual compounds, as shown in other insects^44^. Thus, combinatorial coding of odorant information is key for odour quality perception, and is here supported by the identification of distinct functional types of Ors and OSNs responding to salient human odorants, which translate into the complete percept of host odour detected by the mosquito. From this perspective, it is interesting to observe the differential behavioural responses to the distinct subtractive blends. While these results indicate that a subset of salient odorants contribute more significantly to host attraction than others, future analysis is required to verify this.

### Changes in neural activity, or expression and behavioural valence

Adult maturation in *An. coluzzii* changes the valence of the full synthetic human odour, from aversive to attractive for teneral and host-seeking mosquitoes, respectively. This change is correlated with changes in *Or* expression, including select *Or*s that encode for receptors that are tuned to salient human odorants, and the sensitivity of OSNs tuned to these odorants. Similarly, regulation of peripheral chemosensory receptor gene expression has previously been demonstrated to be linked to an altered sensory response to CO_2_, in *An. coluzzii*^5^ and *Ae. aegypti*^1^, ^Hill *et al*., *submitted*^, and to 1-octen-3-ol in *Ae. aegypti*^1^. Our results thus provide further evidence to support the hypothesis that the sensitivity of the olfactory system of mosquitoes is linked to the relative abundance of gene transcripts.

A blend of phenol, butyl acetate and monoterpenes, while not all uniquely detected by Or1, Or2 and Or75, was sufficient to drive attraction in host-seeking *An. coluzzii*, in the presence of CO_2_. Removal of these compounds from the full synthetic blend made 4 dpe female mosquitoes indifferent to the remaining compounds, emphasizing that these compounds are required and play a critical role in human host recognition. Interestingly, while the transcript levels of *Or1* and *Or2* only appear to change in response to adult maturation, the transcript level of *Or75* decreases significantly in response to blood feeding^20^, at a time when females are behaviourally indifferent to human odour^45^. This merits further analysis of the role of Or75 and the monoterpenes detected by this receptor.

A single *Or*, *Or39*, encoding for a receptor responding to 1-hexanol and sulcatone in human body emanates, was found to significantly decrease in abundance during adult maturation. While 1-hexanol and sulcatone are not exclusively detected by Or39, the removal of these compounds from the full synthetic human odour resulted in a lack of preference of 1 dpe females, whereas 4 dpe females were significantly attracted to the subtractive blend, in the presence of CO_2_. This suggests that the observed preference of 1 dpe females for the control, interpreted here as either avoidance or behavioural inhibition to human odour, may be directly conferred by signalling through Or39, and that the decreased abundance of *Or39* in maturing females lifts this inhibitory signal. Additional support for this was found when testing 1-hexanol and sulcatone as a binary blend in combination with CO_2_, indicating that teneral females are deterred, *i.e*. prefer the control arm, whereas host-seeking mosquitoes are attracted by the blend. The latter may be a result of enhanced receptivity of the olfactory system, not only to these compounds but also to CO_2_^5^. While not sharing any orthologous relationship, AaegOr4 was recently shown to confer sensitivity to sulcatone in *Ae. aegypti*, and to be tightly linked to the preference of human odour^18^. Whether *AaegOr4* is regulated in response to adult maturation, and thereby play a similar role, remains to be revealed.

The olfactory system of female mosquitoes undergoes concerted changes in olfactory gene abundance, which correlate with changes in olfactory sensitivity to human odorants throughout their life cycle^1,5,20,29^^, Hill *et al*., *submitted*^. This allows females to engage in an appropriate, context-dependent behaviour at times when they require blood for egg development^7^. In addition, the observed deterrence of teneral females in response to the synthetic human odour is interesting, and may be seen as an adaptation to decrease the risk associated with blood feeding.

## Conclusion

A combined GC-SSR analysis approach allowed us to identify salient human odorants and the *An. coluzzii* Ors that detect them. This led to the formulation of a blend of 15 Or ligands that is sufficient for both attraction by host-seeking females and aversion in teneral females. Expression analysis implicated key Ors and cognate human odorant ligands that mediate the age-dependent behavioural switch. This strongly supports the hypothesis that host seeking is regulated by changes in *Or* expression and neural sensitivity.

## Material and Methods

### Drosophila stocks

The 49 available UAS-AcolOR transgenes were crossed into the ΔHalo genetic background containing the Or22a-Gal4 construct^13^. All flies courtesy of Prof. John Carlson (Yale University). The functional characteristics of the AcolORs were validated through electrophysiological analysis, as described below, using a subset of the diagnoistic compounds reported by Carey *et al*.^13^ (Supplementary Table 1).

### Mosquitoes

*Anopheles coluzzii* (Suakoko strain) were reared as previously described^5^. For the experiments, non-blood-fed female mosquitoes, either 1 day post-eclosion (dpe) (12**–**24 h) or 4 dpe, were used.

### Collection of human odour

Headspace volatiles from whole human body volatiles were collected as previously described^46^, by placing individual naked volunteers (two groups with each 20 volunteers) in customised heat-sealed cooking bags. Synthetic air was introduced into the bags, and then extracted with pumps through columns containing PorapakQ for 2.5 h. The collection of human odour was performed in accordance with national guidelines and regulations, and the experimental protocol was approved by a member of the Ethics review committee at the Swedish University of Agricultural Sciences. Informed consent for study participation was given by each volunteer. Trapped volatiles were desorbed using pentane, and the volatile collections from each group were then pooled and concentrated under a gentle stream of nitrogen to contain 0.25 minute equivalents µl^−1^. Before concentration, heptyl acetate (1 µg, 99.8%; Aldrich, St. Louis MO, USA) was added to aliquots of each extract as an internal quantification standard.

### Combined GC-SSR and GC-MS analyses

Combined gas chromatography (GC) and single sensillum recording (SSR) analysis was used to characterise the odour-evoked responses of the 49 Ors ectopically expressed in the *Drosophila* ab3A odorant sensory neurons^13^. Recordings were done as previously described^18^. Separation of the volatiles in the collected extracts was done as previously described^12,18^. In brief, an Agilent 6890 GC (Agilent Technology, Santa Clara, CA, USA) fitted with a fused silica capillary column (30 m × 0.25 mm i.d.) coated with a non-polar HP-5 stationary phase (d.f. = 0.25 µm) was used. The GC was fitted with a make-up hydrogen-fed four-way cross at the end of the column, delivering half of the effluent to the flame ionization detector (FID), and the other half to the air stream passing over the antenna of the fly via a transfer line. Responses to eluting compounds were verified through at least three independent injections.

Bioactive compounds were identified as previously described^12,18^. Briefly, we used a combined Agilent 6890 N GC and 5975 mass spectrometer (MS) (Agilent Technology) fitted with the same type of non-polar column, and ran this under the same conditions as for the GC-SSR analysis. The active compounds were identified by comparison with reference mass spectra in our custom made and commercially available libraries (NIST05 and Wiley). These compounds were confirmed by parallel injections of synthetic reference compounds (Supplementary Table 2) and authentic samples on the GC-SSR and GC-MS. To determine the emission rate for each bioactive compound, the absolute quantities, normalised to the internal standard, were divided by the total time over which the respective collections were made to calculate emission rate.

### Single sensillum recordings

Stand-alone SSRs were used to determine the sensitivity of the *Drosophila* ab3A odorant sensory neurons ectopically expressing the *An. coluzzii* Ors, to the GC-SSR-active compounds. In addition, *in vivo* SSRs were performed on three morphological types of trichoid sensilla, TC, TE and TD, on the antennae^29^, which are likely to express the Ors^16^, of 1 dpe and 4 dpe females, using a smaller set of compounds. For the *in vivo* SSR analysis, a characteristic panel of odorants^29^ was used to distinguish the 2 and 4 functional classes of TC and TE sensilla^29^. Serial dilutions of each compound were prepared v/v in hexane, and 15 µl aliquots were loaded into delivery pipettes on the day of recording as previously described. Each pipette was used once. Each trichoid sensillum houses two OSNs, distinguishable by differences in spike amplitude^29^. The response to odour stimuli was analysed by counting the number of spikes present during a 0.5 s prestimulus period and compared with the number elicited during the 0.5 s stimulus delivery period. Spikes were counted off-line using the software package Autospike^TM^ (Syntech, Germany). The outcome was multiplied by 2 to get a spikes s^−1^ measurement. The sensitivity of the trichoid OSNs of 1 dpe and 4 dpe females was analysed using a repeated measures ANOVA (JMP® Pro 13.0.1. SAS Institute Inc., Cary, NC, USA).

### RNA extraction and qPCR

Gene expression assays were conducted to assess the transcript abundance of the functionally characterised *Or* genes in the antennae of 1 dpe and 4 dpe female mosquitoes. For each of the six paired biological replicates, circa 100 mixed-sex pupae were obtained from the same cohort, and separated into different adult rearing cages. The antennae of 1 dpe or 4 dpe female mosquitoes from each treatment group were dissected into 300 µl Trizol (Invitrogen Corporation, Life Technologies, Carlsbad, California, USA) and stored at −80 °C until RNA extraction. The tissue of both treatment groups per replicate were processed side by side until the reverse transcription (RT) step^5^. Total RNA was extracted in 500 µl Trizol reagent (Invitrogen Corporation) following the manufacturer’s protocol. The RNA pellet was washed in 70% ethanol and then in 90% ethanol, dried briefly and re-suspended in 30 µl RNAse free water (Bio-Rad Laboratories, Inc., Hercules, CA, USA) on ice. RNA yield and quality were analysed by absorbance measure (Nanodrop 2000c, Thermo Scientific, Wilmington, Delaware, USA) prior to DNAse treatment. Treatment with TURBO DNAse (Ambion, Life Technologies, Carlsbad, California, USA) was done according to the manufacturer’s protocol. DNAse digestion was stopped by adding TURBO DNAse inactivator (Ambion). The product was immediately used for the RT step using the iSCRIPT reaction mix (Bio-Rad Laboratories) in three technical replicates. A 1:1 mix of oligo dT and random hexamer primers was used, in final volumes of 20 µl each, containing 8 µl of the RNA sample. The three technical replicate cDNA products were then mixed and diluted three times with PCR grade water to obtain the template for subsequent qPCR assays.

### Primer design

Primers, previously used by Iatrou and Biessmann^47^, were tested *in silico* for quality, specificity and secondary structure/primer dimers using Primer 3, implemented in Justbio (www.justbio.com) and Oligoanalyser Software (Integrated DNATechnologies; http://eu.idtdna.com/analyzer/Applications/OligoAnalyzer). Where passed, the primers were used for qPCR assays (Supplementary Table 3). Where desired, or for Ors whose primers were not available, other primers were designed using Primer 3 from available *An. gambiae* genome sequence information (www.vectorbase.org) (Supplementary Table 3). Primers were designed to have a melting temperature (T_m_) of 60 °C, and a product size of 120 to 180 bp; and where possible include an exon within the amplicon or straddle an exon-intron junction so as to exclude genomic DNA from the qPCR products. Three sets of primers were designed for each target, usually in the first two exons to maximise product independent of RT efficiency. The best primer combinations were selected by analysing the specificity, and compatibility of each primer set *in silico* using BLASTn and Oligoanalyser. The best two combinations were tested by qPCR and a selection made by comparing the consistency of amplification in three technical replicates per biological replicate.

Eight reference genes, previously tested for use in a study of *Or* expression in the maxillary palp of *An. coluzzii*^5^ were tested to produce the most stable combinations for this study (Supplementary Table 3). A normalisation factor was selected based on the most stable combination of reference genes under our experimental conditions and tissue (Genex version 5, MultiD Systems, Göteborg, Sweden).

### Quantitative Real-Time PCR

Quantitative PCR was done using the SYBR Green fluorescent dye to detect product accumulation at the end of each cycle, as previously described^5^. The ∆∆Ct method^48^, following the MIQE guidlines^49^, was used to determine changes in gene expression relative to 1 dpe mosquitoes, on Genex Version 5 (Multi D Systems, Sweden). Transcript levels per treatment were normalised to a reference factor comprising the geometric means of the three most stable reference genes, *RpL13*, *RpS4* and *UBQ-1*. Transcription levels were compared between genes per group (1 dpe and 4 dpe) using a two tailed paired Students t-test implemented in Genex v5 after checking data for normality and homogeneity of residuals using Kolmogrov’s test. For non-normalised data, a Mann Whitney U test was used.

### Behavioural analysis

A Y-tube olfactometer (100 mm inner diameter × 1200 mm total length), illuminated from above with red light at 2–5 lx, was used to assess the behavioural response of 1 dpe and 4 dpe mosquitoes to synthetic human odour blends. A charcoal-filtered and humidified laminar air stream (26 ± 1 °C, RH 75 ± 5%) flowed through the olfactometer at 30 cm s^−1^. In the first experiment, a synthetic odour blend, mimicking the composition and ratio of all compounds identified in the human headspace (Fig. 1C,D), was used. The blend was diluted in pentane and released by diffusion from wick dispensers^49^ to allow for the release of all compounds in constant proportions throughout the experiment. As a control, pentane was released in a similar manner. The wick dispensers were inserted into glass wash bottles (500 ml; Lenz Laborglas, Wertheim, Germany), and delivered to the upwind end of the olfactometer through Teflon™ tubes, by passing charcoal-filtered and humidified air, at 0.5 l min^−1^, through the wash bottles. The blend was embedded within a background of pulsed CO_2_ (1200 ppm at 0.5 Hz), used to activate the mosquitoes, as previously described^5^. Preliminary experiments were performed to determine the release rate of the blend eliciting the highest attraction (data not shown)^50^. Subtractive blends (Fig. 3), designed to test the hypothesis that changes in *Or* transcript abundance is linked to the observed shift in host seeking behaviour of 1 dpe and 4 dpe mosquitoes, were tested in the next set of experiments. The subtractive blends maintained the same concentration and release of compounds by making up the volume of missing components with pentane.

Female mosquitoes were kept individually in 6 cm × 10 cm i.d. release cages for 6 h before the experiments. During this acclimatisation period, the mosquitoes were provided with water through a moistened filter paper placed against the mesh, which covered one end of the cage. The release cages were placed at the downwind end of the olfactometer, and the mosquitoes were allowed 5 min to acclimatise, after which the door of the release chamber was opened. Thereafter, the mosquito was given 5 min to make a choice. Mosquitoes that did not move were considered as non-responding and were not included in subsequent analysis. A minimum of 30 single mosquitoes per age group was tested in each experiment. All mosquitoes were tested 0–4 h into the scotophase, the peak activity period of host seeking. For visualisation of the behavioural response, a preference index was calculated as (T − C)/(T + C), where T is the number of mosquitoes associated with the test odours and C the number of mosquitoes associated with the control. Nominal logistic regression was used to analyse the behavioural responses of teneral and host-seeking mosquitoes, in which preference was the categorical response variable and the number of mosquitoes the predictor variable (JMP® Pro 13.0.1. SAS Institute Inc., Cary, NC, USA).

## Supplementary information

Supplementary information

Supplementary Table 1

## Data Availability

The datasets generated during and/or analysed during the current study are either included in this published article (and its Supplementary Information files) or available from the corresponding author on reasonable request.
