# Supplementary material for: Age-dependent regulation of host seeking in Anopheles coluzzii
Source: Sci Rep. 2019 Jul 4;9:9699. doi: 10.1038/s41598-019-46220-w (PMC6609780; doi:10.1038/s41598-019-46220-w)
Supplement: Supplementary file 1 — Supplementary information [file 41598_2019_46220_MOESM1_ESM.pdf]

Age-dependent regulation of host seeking in *Anopheles coluzzii*

Omondi AB, Ghaninia M, Dawit M, Svensson T, Ignell R

Supplementary data

Supplementary figure 1. Relative transcription levels of *Or*s in teneral (1 day post-eclosion) (green) and host-seeking (red) *Anopheles coluzzii* females. Statistical significance was tested using a two tailed paired Students t-test or a Mann Whitney U test. Error bars represent standard errors of the mean. *$p<0.05$, **$p<0.01$.

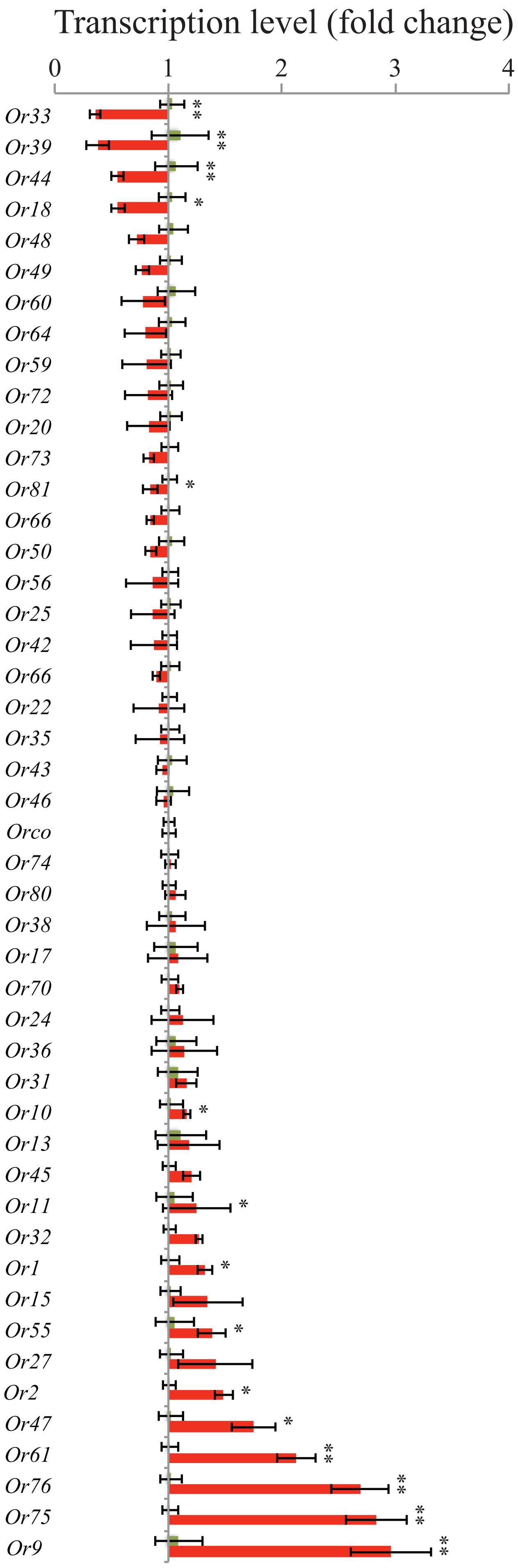

Transcription level (fold change)
0
1
2
3
4
Or33
Or39
Or44
Or18
Or48
Or49
Or60
Or64
Or59
Or72
Or20
Or73
Or81
Or66
Or50
Or56
Or25
Or42
Or66
Or22
Or35
Or43
Or46
Orco
Or74
Or80
Or38
Or17
Or70
Or24
Or36
Or31
Or10
Or13
Or45
Or11
Or32
Or1
Or15
Or55
Or27
Or2
Or47
Or61
Or76
Or75
Or9

Supplementary Table 2. Synthetic chemical compounds used for the electrophysiological and behavioural analyses.

Compound	CAS No	Source	Purity %
1-Hexanol	111-27-3	Sigma	99%
3-Octanol	589-98-0	Sigma-Aldrich	99%
(*R*)-1-octen-3-ol	3687-48-7	Gift from James Logan	99%
2-Nonanol	628-99-9	Sigma-Aldrich	99%
Octanal	124-13-0	Sigma	99%
Nonanal	124-19-6	Acros	95%
Decanal	112-31-2	Sigma	98%
Butyl acetate	123-86-4	Sigma-Aldrich	99.5%
Sulcatone	110-93-0	Sigma	99%
Phenol	108-95-2	Sigma	98%
Benzaldehyde	100-52-7	Sigma	99%
Acetophenone	98-86-2	Acros	99%
Limonene	5989-27-5	Sigma-Aldrich	99%
Linalool	78-70-6	Fluka	97%
α-Terpineol	98-55-5	Sigma-Aldrich	90%

Supplementary table 3. Primers used for the quantitative PCR analysis of odorant receptor gene expression in the antennae of *Anopheles coluzzii*. Product sizes of cDNA and genomic DNA (gDNA) are indicated.

Gene name	Primer	Sequence 5' to 3'	Product (bp)		References
			cDNA	gDNA	
Ribosomal protein S7	AgRpS7F	CACCGCCGTGTACGATGCCA	132	132	Pelletier and Leal, 2011
	AgRpS7R	ATGGTGGTCTGCTGGTTCTT			
Alpha tubulin elongation factor	EF1α-F	TGGAAAGCGTCCTTGTCAG	186	938	Ponton *et al*., 2011, Omondi *et al*., 2015
	EF1α-R	GCCATGCTCCAGACAGTACA			
	AgDLAPF	TTCTCGGATATGCCGATTTC	113	567	Omondi *et al*., 2015
	AgDLAPR	TGGGGCTCCATACTTCAGAC			
TATAA Box binding protein	AgTBPF	CTGTCCGAGATCAGAAAGCAC	151	-	Omondi et al., 2015
	AgTBPR	ATCCACCCATCCAGGTAGAGT			
Ubiquitin	UBQ-F	GCAAGCTAGTAGTGCCGTCTG	190	-	Robertson and Kent, 2009
	UBQ-R:	TCGCGTGTGTGATTTATTCAG			
Ribosomal protein L13	RpL13F:	ATCCTGTCTGGTAACTCGGTG	169	252	Robertson and Kent, 2009
	RpL13R	CCTTCCACAACATACGGCTC			
Ribosomal protein S4	RpS4F:	CGAGGTGACGAAGATTGTGA	124	124	Omondi *et al*., 2015
	RpS4R:	AAGTATTCGCCGGTCTTGTG			
OR Co-receptor	AgOrcoF	GACTATTTCCGGAGCCAAGTTT	88	200	Omondi *et al*., 2015
	AgOrcoR	CAGCACCATGAAGTAGGTGACA			
Odorant receptor Or1	AgOr1F	CGTAATCGGATATTTGTGCTACG	153	281	Iatrou and Biessmann, 2008
	AgOr1R	GTCTAGCAAACAGTCGTCACG			
Odorant receptor Or2	AgOr2F	GTCTGCTGGAGTTCCTGTCGT	164	246	This study
	AgOr2R	CTGCTCCAGTACCTCGTTCG			
Odorant receptor	AgOr3F	GGTGACGGTGGAAACTTACG	150	228	Iatrou and Biessmann, 2008
	AgOr3R	TGGATCGTTGCAGTAGCAG			
Odorant receptor	AgOr4F	ATGCTGCAGTGGTTGACCTG	153	219	Iatrou and Biessmann, 2008
	AgOr4R	AAGCTGCGTCCCGAGTAAAC			
Odorant receptor	AgOr5F	TGGAAACCTACGGATTCTGCTAC	180	253	Iatrou and Biessmann, 2008
	AgOr5R	AAAACTTCCCAGCCGAGATG			
Odorant receptor	Or6F	GGAGTTGATTGGGTACGAAGG	181	560	Iatrou and Biessmann, 2008
	Or6R	TACGCACACAGCATCAGCAG			
Odorant receptor	AgOr9F	GGTAATGTACTTGGTGTGCCTTG	174	-	Iatrou and Biessmann, 2008
	AgOr9R	CCTCGAGCGGTTATTTGTTG			
Odorant receptor	Or10F	GGTGACCTACATCTGCCTGATAG	172	240	Iatrou and Biessmann, 2008; this study
	Or10R	CCGCACCTCGTTCGAGTGCCAGTAG			
Odorant receptor	AgOr11F	TGCTACATTACCACGATGACGAC	145	232	Iatrou and Biessmann, 2008
	AgOr11R	TCTGTAGCATCCGGTTCGTG			
Odorant receptor	AgOr13F	AGAAACCATTGCTTACTGCTATTTTGG	176	248	Iatrou and Biessmann, 2008
	AgOr13R	AGCAAAACTTGCCAGCAGTG			

Odorant receptor	AgOr14F	TGGAACGCGCCTCTCTAC,	169	257	Iatrou and Biessmann, 2008
	AgOr14R	CAGCTCCACATCGACGAAAC			
Odorant receptor	AgOr15F	AACGGAAACCTTTGCCTACTG,	166	244	Iatrou and Biessmann, 2008
	AgOr15R	AGCAGTGATGCCAACTGGAC			
Odorant receptor	AgOr16F	CTTGGGTAATCAACTTTCTGACG	130	193	Iatrou and Biessmann, 2008
	AgOr16R	AACAGACCGTTGAGCACGTAG			
Odorant receptor	AgOr17F	AACGGAAACCTTTGCCTACTG	175	250	Iatrou and Biessmann, 2008
	AgOr17R	AAATTTGCCAGCGGTAATGC			
Odorant receptor	AgOr18F	TACTCGTACCAGCCGGAACAG	116	178	Iatrou and Biessmann, 2008
	AgOr18R	CAACCTCAAATGCCATC			
Odorant receptor	AgOr21F	AGAAGCCATCGGTCGAG	136	209	Iatrou and Biessmann, 2008
	AgOr21R	GAAGCACCAGGTCAGAATGC			
Odorant receptor	AgOr22F	GAGCAGCATGCCGTATGAAC	156	224	Iatrou and Biessmann, 2008
	AgOr22R	GAAATCCACACCGGACTTTCTC			
Odorant receptor	AgOr23F	ATTGGGAACATTCCTATCATC	153	222	Iatrou and Biessmann, 2008
	AgOr23R	GGTGAGAACCCATCGGAG			
Odorant receptor	AgOr24F	CTATTGTGTTTCTTGGATCGGTTC,	110	166	Iatrou and Biessmann, 2008
	AgOr24R	GATGGCACCAATTTTCAACG			
Odorant receptor	AgOr25F	GAGCTGCGGAACGAAACG	166	285	Iatrou and Biessmann, 2008
	AgOr25R	TCGGGATCAGCTCGAACATC			
Odorant receptor	AgOr26F	TGTATGTCTTGGAATGGTAGTGCTG	165	224	Iatrou and Biessmann, 2008
	AgOr26R	TTGAAAATCCCATCCGCTAC			
Odorant receptor	AgOr27F	GCCTGTCGATGGTTGTTTTG	122	180	Iatrou and Biessmann, 2008
	AgOr27R	GAGCTCAGTGCCGAGGTAAG			
Odorant receptor	AgOr29F	TCTGATGATGTTCTACATTTTGCTG	126	186	Iatrou and Biessmann, 2008
	AgOr29R	TTATCTGTCAGCTGCGTTCC			
Odorant receptor	AgOr30F	GGTGCCTCATGCTGTTCTACATAC	136	211	Iatrou and Biessmann, 2008
	AgOr30R	TCTGCGTTTGAGGTGAACTG			
Odorant receptor	AgOr31F	GCTACTATGGATCACAGCTCTACTACG	133	213	Iatrou and Biessmann, 2008
	AgOr31R	ACGGTTTCTTCGCTCTCATC			
Odorant receptor	AgOr32F	CTACCACGGGTCAGCTATCAG	177	236	Iatrou and Biessmann, 2008
	AgOr32R	TTCAGTGTCGGCAAGGATG			
Odorant receptor	AgOr33F	CTTCTTCGGCAACCGTGTC	171	256	Iatrou and Biessmann, 2008
	AgOr33R	TCATACGTCAGCGGAAACAG			
Odorant receptor	AgOr34F	GCTTCGGCTTATCAGGTAGC	173	252	Iatrou and Biessmann, 2008
	AgOr34R	TACCAGCGCGAGGAGTAGATG			
Odorant receptor	AgOr35F	ACGGTTCCATCATGTACGATG	154	241	Iatrou and Biessmann, 2008
	AgOr35R	GCCTTTATGAAGCCACCTTTG			
Odorant receptor	AgOr36F	CTGGTCGATCGGCTCAATG,	160	245	Iatrou and Biessmann, 2008
	AgOr36R	CGAGCGACTTCTGCGTCTTAC			
Odorant receptor	AgOr37F	CGTCCGCATTCAAGACATC	184	264	Iatrou and Biessmann, 2008
	AgOr37R	AATCCCATCTCGTACCAACG			

Odorant receptor	AgOr38F	GCACGGTTGTGCGAGAATC	167	236	Iatrou and Biessmann, 2008
	AgOr38R	GAAGGTAGCCATCGACGTTTG			This study
Odorant receptor	AgOr39F	TACAGCGGCACAATCATCC	126	202	Iatrou and Biessmann, 2008
	AgOr39R	GTACTGCGCCCTAATCATCATC			
Odorant receptor	AgOr40F	TTGGTCAGACCCTCAAGAACC	123	226	Iatrou and Biessmann, 2008
	AgOr40R	GTCGTTGAGCATTCATCAGG			
Odorant receptor	AgOr41F	CGTTATCACACTGTTCGTATTGC	168	255	Iatrou and Biessmann, 2008
	AgOr41R	GACAGCTCGTTCCAGGTGAG			
Odorant receptor	AgOr42F	CGTTGAACGAACTGCATCG	173	244	Iatrou and Biessmann, 2008
	AgOr42R	CTGTTCCGGTAGGTCCATCTC			
Odorant receptor	AgOr43F	AGCTATGTCTGCTAGGAACTATCCTC	157	222	Iatrou and Biessmann, 2008
	AgOr43R	CTGGCAACAGTCATTTCCTTG			
Odorant receptor	AgOr44F	GAGCTATGTCTGCTAGGAACTATCC,	149	218	Iatrou and Biessmann, 2008
	AgOr44R	GTCATTTCCATGGCATGTTG			
Odorant receptor	AgOr45F	CGGCTGAAGGACAGTGTG	168	234	Iatrou and Biessmann, 2008
	AgOr45R	AGAATAGGCCGCTCGGATG			
Odorant receptor	AgOr46F	GGGGAACGAGCTGACGTTG,	121	175	Iatrou and Biessmann, 2008
	AgOr46R	CTGTTGGCTTTGCTGTATTATTG			
Odorant receptor	AgOr47F	TGGGGAACGAACTGACTTTG,	125	180	Iatrou and Biessmann, 2008
	AgOr47R	TCGCTGTTGGCTTTGCTG			
Odorant receptor	AgOr48F	GGAAACCAGCTCACGGAAG,	182	254	Iatrou and Biessmann, 2008
	AgOr48R	ATAAGTTGCGCGAACGTCAC			
Odorant receptor	AgOr49F	TTCGGCAATAGGCTGTCG	115	173	Iatrou and Biessmann, 2008
	AgOr49R	CGTGACGGATCATCTGTTGG			
Odorant receptor	AgOr12/50F	AAATGCATCAGCTGGCAATAC	148	223	Iatrou and Biessmann, 2008
	AgOr12/50R	ATCATTGCCGTTACGTCCAG			
Odorant receptor	AgOr51F	TTGGAACGCGTCTCTCTACCC,	172	257	Iatrou and Biessmann, 2008
	AgOr51R	XXXGTTCCATATCGACGAAGC			
Odorant receptor	AgOr52F	TACTACTGCTGCAAGCTCGTTG	155	240	Iatrou and Biessmann, 2008
	AgOr52R	GCGAGCAGTATGATGCTGAG			
Odorant receptor	AgOr53F	CGATCGAAACCTACGCCTACTG,	150	209	Iatrou and Biessmann, 2008
	AgOr53R	GTGTTTGCGAGCGTCGTATC			
Odorant receptor	AgOr54 F	TTGGGAGAAACGTTCAACATC	149	220	Iatrou and Biessmann, 2008
	AgOr54R	CCAAAGCCATACGAAAGTCG			
Odorant receptor	AgOr55F	CTCGGTACTCAACTTTCGATGG	150	223	Iatrou and Biessmann, 2008
	AgOr55R	AAACTTGCCGGCGGTTATAC			
Odorant receptor	AgOr56F	CTGGGTATGGTCGTGCTGTC,	158	569	Iatrou and Biessmann, 2008
	AgOr56R	TGGAAAATTGCATCCGCTAC			
Odorant receptor	AgOr58F	TGGGTTGTGTGATTTGCTCAAG	137	197	Iatrou and Biessmann, 2008
	AgOr58R	TGAGTATGCTGGACCGGATG			
Odorant receptor	AgOr59F	GCGGCATTGTGCAATTTTC,	173	144	

	AgOr59R	CGACCGAGATGGATACTC			Iatrou and Biessmann, 2008
Odorant receptor	AgOr60F	CTGTCGTATCTTGTCTCAAATTAACG	174	255	Iatrou and Biessmann, 2008
	AgOr60R	CTGAACGATAGCGGCTTCTG			
Odorant receptor	AgOr61F	CGCGGAATCGAGAAGTTAAATG,	185	257	Iatrou and Biessmann, 2008
	AgOr61R	AGGCCTCCGTACGAACACTG			
Odorant receptor	AgOr62F	GTGGAAGATTTGAACG,	153	236	Iatrou and Biessmann, 2008
	AgOr62R	GTTGGGACTGCAAAATCACG			
Odorant receptor	AgOr63F	CCACGGAGTGGAAAATTTAAGAC	182	243	Iatrou and Biessmann, 2008
	AgOr63R	CGGCGAAGCTGAATTGTATC			
Odorant receptor	AgOr64F	GCAAAAGCATCGATAGTGTTAATG,	120	209	Iatrou and Biessmann, 2008
	AgOr64R	GTCGCAGTGTTTCGGTTGAC			
Odorant receptor	AgOr65F	TGTTACTTTGGCAATGAAATCAAC	126	-	Iatrou and Biessmann, 2008
	AgOr65R	AGCAGTGATCTGCTGAAATGC			
Odorant receptor	AgOr66F	CGACCGTATTCAATCTATTTTTGG,	163	344	Iatrou and Biessmann, 2008
	AgOr66R	ACCAGGGCAGATTCGAGATG			
Odorant receptor	AgOr68F	TGTGCTAGGGGCTATCATCGAG	151	263	Iatrou and Biessmann, 2008
	AgOr68R	ACCAGCAATCGACATTCCAG			
Odorant receptor	AgOr69F	TTGTGATTGGACATTTTG	142	206	Iatrou and Biessmann, 2008
	AgOr69R	GTTAACATCATGGGGCGTTG			
Odorant receptor	AgOr70F	GGATTGGTATCCTACATACTCGTTC	189	245	Iatrou and Biessmann, 2008
	AgOr70R	GGCATTGCTGTCTGCTCATC			
Odorant receptor	AgOr71F	TACTCGTCTGTCGTTATAGGCTACT,	185	248	Iatrou and Biessmann, 2008
	AgOr71R	GTGCATGTCCATCCTTATACAACCC			
Odorant receptor	AgOr72F	GGTTCTTTGTGATTGGACATTTGG,	169	235	Iatrou and Biessmann, 2008
	AgOr72R	ATGGGATGAAAGCCATACGC			
Odorant receptor	AgOr73F	AACTATCAGGGTTCTTTGTGATTGG	151	214	Iatrou and Biessmann, 2008
	AgOr73R	ATCATCGGACATTGCTGACG			
Odorant receptor	AgOr74F	CCAACATATTCCTACCTGTATGTGATG	184	250	Iatrou and Biessmann, 2008
	AgOr74R	TCATCGGGTTTTGCTGTCTG			
Odorant receptor	AgOr75F	ATGTGCATGCTGGTGGAGATGCTG	187	5974	Iatrou and Biessmann, 2008, modified
	AgOr75R	AAACAGCTCGGCCGATACC			
Odorant receptor	AgOr76F	CGTGGCCACCTTTATGACG	167	232	Iatrou and Biessmann, 2008
	AgOr76R	CGTACAAGCTGGTGCTGACC			

References

Pelletier, J., & Leal, W. S. (2011). Characterization of olfactory genes in the antennae of the Southern house mosquito, *Culex quinquefasciatus*. *Journal of insect physiology*, *57*(7), 915-929.

Iatrou, K., & Biessmann, H. Sex-biased expression of odorant receptors in antennae and palps of the African malaria vector *Anopheles gambiae*. *Insect Biochem. Mol. Biol.* **38**, 268-274 (2008).

Omondi, B. A., Majeed, S., & Ignell, R. Functional development of carbon dioxide detection in the maxillary palp of *Anopheles gambiae*. *J. Exp. Biol.* **218**, 2482-2488 (2015).

Ponton, F., Chapuis, M. P., Pernice, M., Sword, G. A., & Simpson, S. J. Evaluation of potential reference genes for reverse transcription-qPCR studies of physiological responses in *Drosophila melanogaster*. *J. Insect Physiol.* **57**, 840-850 (2011).

Robertson, H. M., & Kent, L. B. Evolution of the gene lineage encoding the carbon dioxide receptor in insects. *J. Insect Sci.* **9**, 1-14 (2009).
